# The Potential Role of Circulating Long Miscellaneous RNAs in the Diagnosis and Prognosis of Hepatitis C Related Hepatocellular Carcinoma

**DOI:** 10.3390/ncrna9050062

**Published:** 2023-10-11

**Authors:** Shimaa Abdelsattar, Sally A. Fahim, Hala F. M. Kamel, Hiba Al-Amodi, Zeinab A. Kasemy, Fatma O. Khalil, Mahmoud S. Abdallah, Hanan M. Bedair, Abdel-Naser Abdel-Atty Gadallah, Aliaa Sabry, Mohamed A. Sakr, Mahmoud Selim, Eman M. Abd El Gayed

**Affiliations:** 1 Clinical Biochemistry and Molecular Diagnostics Department, National Liver Institute, Menoufia University, Shebin El-Kom 32511, Egypt; 2 Biochemistry Department, School of Pharmacy, Newgiza University (NGU), Cairo 94114, Egypt; sallyatef@hotmail.com; 3 Medical Biochemistry and Molecular Biology Department, Faculty of Medicine, Ain Shams University, Cairo 11566, Egypt; hfkamel@uqu.edu.sa; 4 Biochemistry Department, Faculty of Medicine, Umm Al-Qura University, Makkah 21955, Saudi Arabia; hsamodi@uqu.edu.sa; 5 Department of Public Health and Community Medicine, Faculty of Medicine, Menoufia University, Shebin El-Kom 32511, Egypt; zeinabkasemy@yahoo.com; 6 Clinical Microbiology and Immunology Department, National Liver Institute, Menoufia University, Shebin El-Kom 32511, Egypt; fatma.khalil@liver.menofia.edu.eg; 7 Clinical Pharmacy Department, Faculty of Pharmacy, University of Sadat City (USC), Sadat City 32897, Egypt; mahmoud.samy@fop.usc.edu.eg; 8 Clinical Pathology Department, National Liver Institute, Menoufia University, Shebin El-Kom 32511, Egypt; hananbedair@yahoo.com; 9 Internal Medicine Department, Faculty of Medicine, Menoufia University, Shebin El-Kom 32511, Egypt; ahmed_naser2004@yahoo.com; 10 Department of Hepatology and Gastroenterology, National Liver Institute, Menoufia University, Shebin El-Kom 32511, Egypt; aliaasabry@liver.menofia.edu.eg; 11 Medical Microbiology and Immunology Department, Faculty of Medicine, Suez University, Suez 43512, Egypt; msakr.md@gmail.com; 12 Internal Medicine Department, Faculty of Medicine, Tanta University, Tanta 31111, Egypt; mahmoud.saleem@med.tanta.edu.eg; 13 Medical Biochemistry and Molecular Biology, Faculty of Medicine, Menoufia University, Shebin El-Kom 32511, Egypt; nagarahmed@yahoo.com

**Keywords:** ribonucleic acids, noninvasive biomarker, progression, hepatitis C, hepatocellular carcinoma, overall survival C

## Abstract

Ribonucleic acids (RNAs) are important regulators of gene expression and crucial for the progression of hepatocellular carcinoma (HCC). This study was designed to determine the diagnostic and prognostic utility of the circulating long miscellaneous RNAs; LINC01419, AK021443, and AF070632 in HCV-related HCC patients. Real-time PCR was used to measure their relative expression levels in the plasma of 194 HCV patients, 120 HCV-related HCC patients and 120 healthy controls. LINC01419 and AK021443 expression levels had significantly increasing linear trend estimates while AF070632 was dramatically downregulated in HCC compared to HCV. Interestingly, LINC01419 and AK021443 served as more significant diagnostic biomarkers for HCC than AF070632 and AFP. Multivariate analysis with cox regression revealed that the high expression of AK021443 [HR = 10.06, CI95%: 3.36–30.07], the high expression of LINC01419 [HR 4.13, CI95%: 1.32–12.86], and the low expression of AF070632 [HR = 2.70, CI95%: 1.07–6.81] were significant potential prognostic factors for HCC. Besides, the Kaplan–Meier analysis showed that HCC patients with high LIN01419 and AK021443 and low AF070632 expression levels had shorter OS. The circulating LINC01419 and AK021443 can be used as noninvasive potential biomarkers for diagnosis and prognosis of HCV-related HCC patients than AF070632 providing new targets for limiting the progression of the disease.

## 1. Introduction

Hepatitis C virus (HCV) infection is the main reason behind hepatic illnesses worldwide. Although the risk attributed to HCV infection has considerably decreased with antiviral drugs, people with cirrhosis are still regarded to be at high risk for developing hepatocellular carcinoma (HCC) even after HCV clearance [1]. Unfortunately, patients infected with HCV are at seventeen times the risk of HCC caused by liver cirrhosis [2,3].

HCC is ranked fifth amongst all types of cancers regarding its prevalence. It is the third leading cause of death from cancer with about fifty thousand new cases yearly [4]. The absence of symptoms in the early stages results in a very high mortality rate. This issue is more prominent in developing countries due to poor screening tools [5].

Patients with HCC may have high levels of alpha fetoprotein (AFP). Studies show a sensitivity of just 60% at a threshold of 20 ng/mL [6,7] with a controversial role for the usefulness of serum AFP in detecting HCC [8].

Epigenetics, encompassing noncoding RNA regulation, DNA methylation, and histone modification, are related to the initiation and progression of HCC including dysregulated signaling pathways in HCC [9,10]. Many vital genes depicted to be associated with HCC pathogenesis were considered vital biomarkers for early HCC diagnoses and prognosis [11,12]. However, in addition to ~20,000 genes that code for proteins, our transcriptome includes a huge amount of long RNAs that do not code for proteins called long noncoding RNAs (lncRNAs) [13].

Several lncRNAs were reported to have an important role in tumorigenesis and metastasis of HCC as highly upregulated in liver cancer (HULC), metastasis-associated lung adenocarcinoma transcript 1 (MALAT1) and HOX antisense intergenic RNA (HOTAIR) [14]. Unfortunately, these RNAs were shown to be nonspecific for HCC [15]. On the other hand, LINC01419, AK021443 and AF070632 showed great uniqueness in HCC resulting from HCV, but not in HCC induced by other causes [16,17].

LINC01419 is a long intergenic non-protein coding of 1521-nucleotide long and located on human chromosome 8 loci 21. On the other hand, AK021443 is an mRNA of 1619-nucleotide long encoding for HELLS; a helicase and is located on human chromosome 10 loci 23. Both LINC01419 and AK021443 have been shown to regulate cell cycle genes. Whereas AF070632 is a clone of mRNA sequence with 1446 bp and located on the short arm of chromosome 1 at position 1p13.2 and surrounded by 64 differentially expressed protein-coding genes [18].

Biomarkers can be executed easily, repeated shortly, and consequently would lead to high participants’ adherence [19]. We, therefore, hypothesized diagnostic and prognostic roles for LINC01419, AK021443 and AF070632 expressions in the plasma as non-invasive biomarkers for HCV-related HCC patients in comparison to HCV patients and healthy controls.

## 2. Results

**Figure 1:**
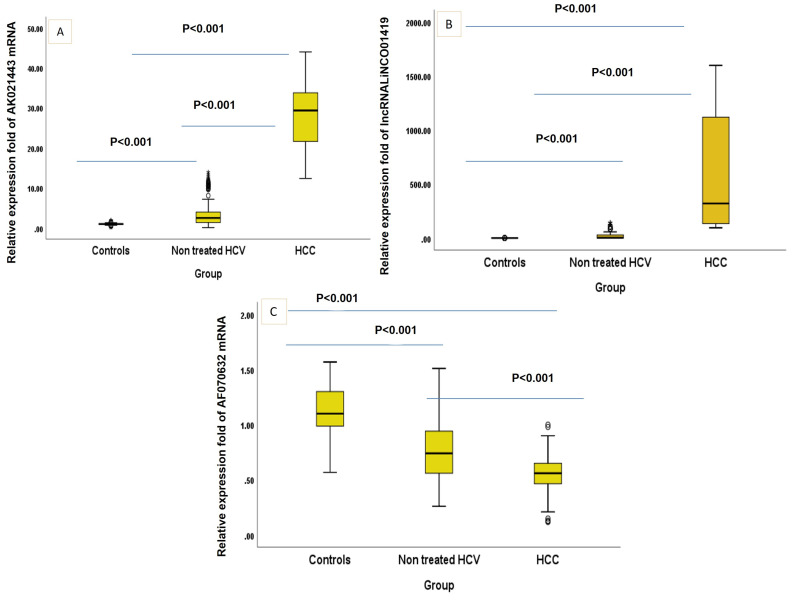
(**A**–**C**). Box-Plot figure of the studied markers. (*p* value was <0.001 between all the studied groups in every single marker).

The demographic characteristics and the laboratory parameters of healthy controls, non-treated HCV and HCV-related HCC patients participating in the study were defined in (Table 1) where age and sex did not significantly differ between the three studied groups. All other parameters showed a significantly increasing linear trend estimate except for BUN. LINC01419 and AK021443 showed significantly increasing linear trend estimates (0.696 [0.663–0.727] and 0.688 [0.654–0.720]) with *p* < 0.001, while mRNA AF070632 showed a highly significant decreasing trend (−0.553 [(−0.595)–(−0.509)] (*p* < 0.001) (Figure 1).

According to Child Pugh classification, AK021443 and LINC01419 expression levels were significantly higher in Child Pugh class A and B (*p* < 0.001) of HCC patients than those of HCV, while mRNA AF070632 was significantly lower in both Child Pugh classes A (*p* = 0.026) and B (*p* = 0.001) in HCC group compared to HCV group (Table 2).

**Table 2:** The expression level of AK021443, LINC01419 and AF070632 according to Child Pugh classes of the studied patients.

	Child Pugh Class	No	Non Treated HCV	No	HCC	Mann–Whitney Test	*p* Value
			Median (IQR)		Median (IQR)		
mRNA AK021443	A	167	2.65 [1.38–5.03]	16	20.52 [13.73–38.38]	6.47	<0.001 * -
	B	27	2.15 [1.30–2.64]	82	28.11 [22.01–33.03]	7.77	
	C	0	-	22	33.84 [27.21–38.65]	-	
LncRNA LINCO01419	A	167	4.07 [1.65–31.05]	16	234.92 [135.69–570.74]	6.48	<0.001 *
	B	27	2.68 [1.60–10.17]	82	215.20 [131.33–1120.55]	7.77	<0.001 *
	C	0	-	22	1101.63 [27.03–1240.10]	-	-
mRNA AF070632	A	167	0.75 [0.56–0.95]	16	0.61 [0.46–0.74]	2.23	0.026 *
	B	27	0.71 [0.55–0.90]	82	0.56 [0.47–0.65]	3.39	0.001 *
	C	0	-	22	0.55 [0.34–0.57]	-	-

**Figure 2:**
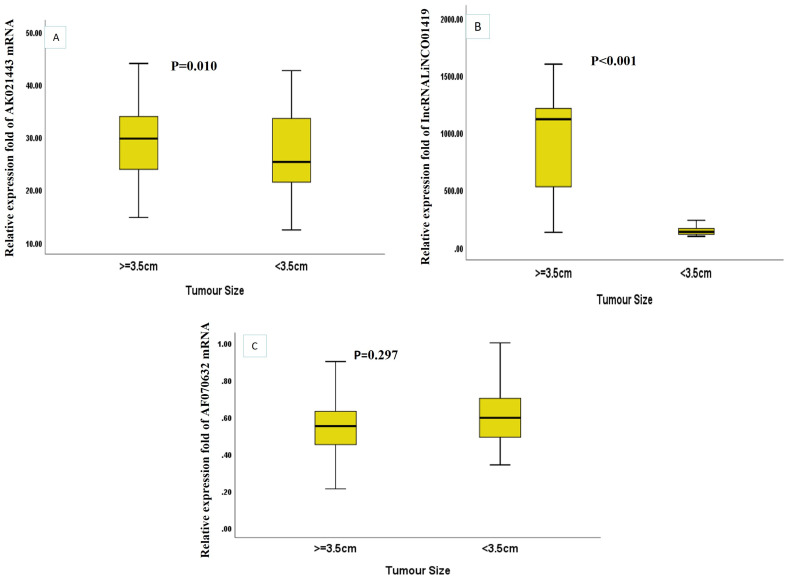
The relative expression levels of the three miscellaneous RNAs in HCC patients according to the tumor size (**A**) AK021443 (**B**) LINC01419 (**C**) AF070632.

* significant regarding the expression levels of the three RNAs in HCC patients according to tumor size, LINC01419 was significantly higher in HCC patients with tumor size ≥ 3.5 cm (*p* < 0.001) while AK021443 was significantly lower in HCC patients with tumor size ≥ 3.5 cm (*p* = 0.010) (Figure 2A–C).

Notably, AK021443s was significantly and negatively correlated with ALT (rs = −0.291), AST (rs = −0.224), D. Bilirubin (rs = −0.224), and positively correlated with viral load (rs = 0.567) while LINC01419 was significantly and positively correlated with AFP and Child Pugh (rs = 0.456 and 0.278, respectively) and negatively correlated with mRNA AF070632(rs = −0.314). Finally, AF070632 significantly and negatively correlated with platelets, AFP, albumin, T. Bilirubin, and Child Pugh score. (rs = −0.230, −0.228, −0.232, −0.245, −0.184, respectively) (Table 3).

**Figure 3:**
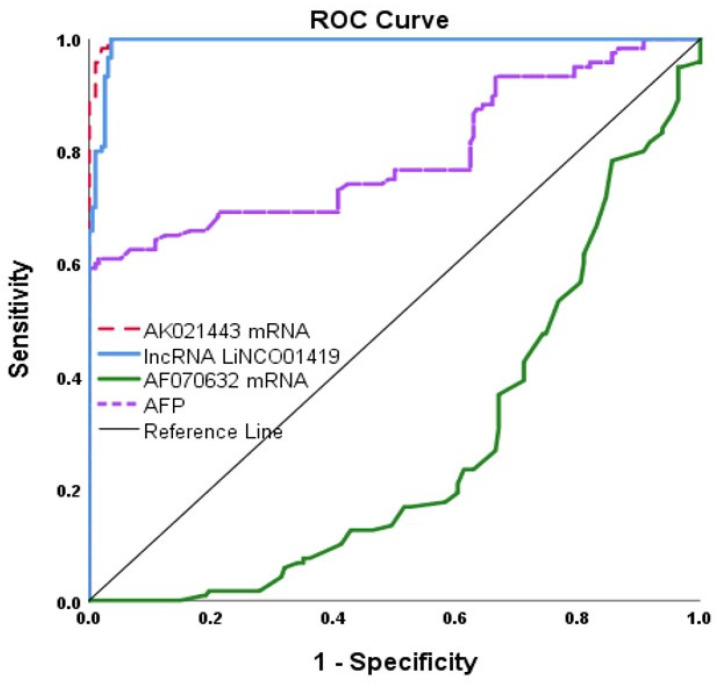
ROC Curve for the three miscellaneous RNAs and AFP.

The expression levels of LINC01419 and AK021443 served as significant diagnostic biomarkers for HCC in HCV-infected patients through ROC curve analysis. The cut of value, AUC, sensitivity, and specificity were >91.84, 0.993, 100% and 97% for LIC01419 and >12.97, 0.998, 100% and 97% for AK021443, respectively. On the other hand, mRNA AF070632 showed a lower AUC of 0.725 at the cutoff point ≤ 0.68, with sensitivity and specificity of 81% and 60%, respectively, compared to LINC01419 and AK021443 when discriminating HCC patients from HCV patients. AFP had low sensitivity of 73% and specificity of 59% (Table 4 and Figure 3).

In univariate analysis, the high expression of AK021443, Child Pugh, the high expression of LINC01419, the low expression of mRNA AF070632 and the high expression of AFP were significantly associated with low Overall survival in HCC patients, Multivariate analysis with Cox regression for clinicopathologic characteristics and the biomarkers was performed revealing that the high expression of AK021443 [HR = 10.06, CI95%: 3.36–30.07], Child Pugh score [HR = 9.97, CI95%: 1.96–50.58], the high expression of LINC01419 [HR 4.13, CI95% 1.32–12.86], the low expression of mRNA AF070632 [HR = 2.70, CI95% 1.07–6.81] and Portal invasion [HR = 2.20, CI95% 1.05–4.60] were significantly potential prognostic factors for HCC (Table 5).

**Figure 4:**
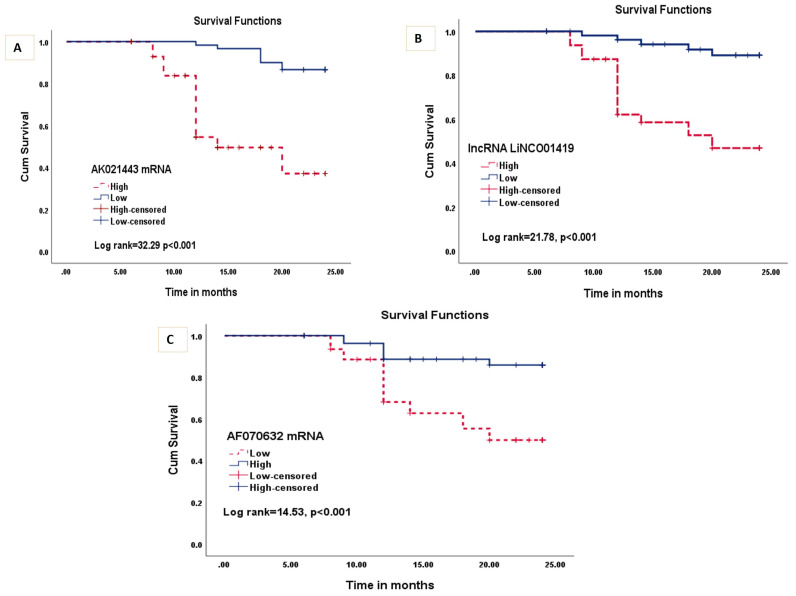
Kaplan–Meier and Log-rank survival tests for (**A**) AK021443, (**B**) LINC01419, and (**C**) AF070632.

Furthermore, Kaplan–Meier and Log-rank survival tests showed that higher expression levels of AK021443 (Figure 4A) and LINC01419 (Figure 4B) in HCC patients, were associated with shorter overall survival (OS) (*p* = 0.001). While the decreased expression levels of mRNA AF070632 were associated with shorter OS (*p* = 0.001) (Figure 4C).

## 3. Materials and Methods

### 3.1. Population of the Study

A case-control study recruiting 120 healthy controls and 314 patients (194 HCV patients and 120 HCV-related HCC patients) at the outpatient clinic of the National Liver Institute, Menoufia University, Egypt, was conducted from March 2022 to March 2023. All patients tested positive for HCV antibody and negative for both HBV surface antigen and HBV core antigen (HBcIg) for exclusion of HBV/HCV co-infected patients. Patients were identified by imaging (Abdominal ultra-sound and triphasic computed tomography) according to the American Association for the Study of Liver Diseases (AASLD) and the European Association for the Study of Liver (EASL) [20] and serum AFP level. Tumor features (number and dimensions of lesions and portal venous invasion) were recorded. Child Pugh score, BCLC stage and MELD score were also calculated. Liver cirrhosis was classified consistent with the Child score classification. Patients were classified into Child-Pugh grades A (5–6 points), B (7–9 points), or C (10–15 points). Patients were also classified according to the BCLC staging system to define tumor extent and liver function impairment. Patients who had liver transplantation, or ever received any treatment for HCV or HCC, had any other type of cancer, or presented with renal insufficiency were excluded from the study. One hundred and twenty healthy with age and sex-matched controls volunteered for the study. They all had completely normal liver function tests and an abdominal ultrasound, with negative results for both viral liver and autoimmune diseases.

Verbal consent was obtained from all participants. The approval for the study protocol was obtained from the ethics committee of the National Liver Institute, Menoufia University (IRB number = 00335\2022) and agreed with the ethical strategies of the 1975 Helsinki Declaration.

### 3.2. Blood Sampling and Laboratory Analyses

Approximately ten milliliters of venous blood were withdrawn from the cubital vein of each participant and divided as follow: two milliliters were transferred into citrated tubes for assessing prothrombin time and INR by Sysmex CS-1600 Automated hemostasis testing (Sysmex Corporation, Kobe, Japan), two milliliters of blood were added to a tube containing EDTA for CBC assay performed by Sysmex XT-1800i automated hematology analyzer (Sysmex, Kobe, Japan), three milliliters of blood were added to another EDTA container, centrifuged and the resulting plasma was kept for total RNA extraction step using miRNeasy kit and finally, three milliliters were placed into plain serum tube, where serum was isolated from clotted blood by centrifugation and immediately placed into eppendorfs and kept at −20 °C for subsequent biochemical analysis of hepatic and kidney function tests as CEA and AFP using Cobas c501 Auto analyzer (Roche, Mannheim, Germany) and virological screening (HCV antibodies, HBsAg, HBcAb, HIV antibodies).

### 3.3. Extraction of Total RNA and cDNA Formation

Extraction of the entire RNA was conducted by using RNeasy plus Universal Kit (QIAGEN, Germantown, ML, USA). Nano-Drop instrument (Thermo Scientific, Waltham, MA, USA) was used to determine the harvest and purity of RNA. The extract was then kept at −80 °C. Using the SensiFAST cDNA Synthesis Kit, Bioline, Germany, we manufactured the cDNA. We used a final volume of 20 µL; 1 µL of reverse transcriptase enzyme, four µL of the Buffer, 10 µL of RNA template and 5 µL of nuclease-free water. A 2720 thermal cycler, Applied Bio systems (Singapore) was adjusted for one cycle in this way: 10 min at 42 °C, 5 min at 95 °C and lastly for 5 min at 4 °C. cDNA produced was stored at −20 °C till real-time PCR (qPCR) step. Measurement of LINC01419, AK021443 and AF070632 genes by quantitative polymerase chain reaction. qPCR step was achieved using SensiFASTTM SYBR Lo-ROX Kit, Columbia, SC, USA. The 20 µL final volume contained 10 µL of SYBR green Master Mix, 1 µL of Nuclease-free water, 6 µL of template cDNA and 1.5 µL of each primer (sense and antisense).

We used the subsequent oligonucleotide primers as previously described [18,21]: 5′-GAAACTCCGAACACATCTG-3′ (sense), 5′-TTCTCCTGCTGGTTGATT-3′ (antisense) for LINC01419, and 5′-CTTGAACCCAGAAGACAGG-3′ (sense) and 5′-ATGGAACATTAGAGGTAGCAC-3′ for AK021443 (antisense) and 5′-CAGGGTGGTGACGTGGGGGA-3′ (sense) and 5′-TGCAGTTAGTCCTGAGCTTGGCA-3′ for AF070632 (antisense). Finally, 5′-TCAAGGCTGAGAACGGGAAG-3′ (sense), 5′-GTGAAGACGCCAGTGGACT-3′ (antisense) for GAPDH as an internal control.

The adjusted conditions for amplification were as follows: initial activation phase at 95 °C for 5 min followed by 45 cycles at 95 °C for 20 s; 60 °C for 30 s; 72 °C for 1 min and a final extension phase at 72 °C for 10 min using 7500 ABI PRISM (Applied Biosystems, Waltham, MA USA) v.2.0.1. The relative quantification (RQ) was then calculated by 2^−ΔΔCT^ method [22] using GAPDH as an endogenous housekeeping gene.

### 3.4. Bioinformatics Analysis

The association between LINC01419, AK021443 and AF070632, and HCV-induced HCC was investigated using the long miscellaneous RNAs disease database (http://www.cuilab.cn/lncrnadisease, accessed on 1 October 2022) and lncRNA Disease V2.0 [23]. By analyzing the data of the expression level of the chips uploaded to the GEO database using GEO2R, the expression levels of LINC01419 and AK021443 are significantly different in different tissues and were low in normal controls compared to HCV-induced HCC patients, while AF070632 showed different expression levels between metastatic and non-metastatic HCC lesions. Although LINC01419 and AK021443 show the gene co-expression mode, there is no crosstalk relationship between them. The protein target of LINC01419 and the expression changes of these LINC01419-regulated targets were added as a Supplementary Materials.

### 3.5. Sample Size

According to Austin and Steyerberg [24] the concept of an event per variable (EPV) of 20 is acceptable for Cox regression. Cox regression involves only independent variables with a large effect size remaining in the result [25,26]). Therefore, an EPV of 11 was relevant in the case of medium to large effect size.

### 3.6. Statistical Analysis

Analyses were performed using SPSS version 28.0 [SPSS Inc., Chicago, IL, USA]. A Shapiro–Wilk test as one of the normality tests was conducted to ascertain the normality of distribution. One-way ANOVA was used to compare the studied groups’ normally distributed data. The homogeneity test was performed to test the equality of variance and to determine the post hoc test following One-way ANOVA where the Tukey test and Tamhane tests were applied accordingly. A Kruskal–Wallis test was performed for not normally distributed variables followed by a Mann–Whitney test to determine the significance between the individual groups. Mann–Whitney was applied for not normally distributed variables between two groups. The chi-square test was used to analyze the qualitative variables like sex, smoking, diabetes mellitus, and hypertension. Linear trend analysis using the Jonckheere–Terpstra test was applied to detect whether there was an increasing or decreasing trend across the ordered groups. The Mann–Kendall [M–K] test was used to detect the presence of linear or non-linear trends [steadily increasing/decreasing or unchanging] in a series of data by estimating the effect size following the Jonckheere–Terpstra [J–T] Test. ROC curve analysis is applied to estimate sensitivity, specificity, accuracy, positive predictive value, and negative predictive value. Spearman correlation was used to assess the strength and direction between the studied markers. The Kaplan–Meier curve was illustrated followed by Cox regression analysis to detect the independent predictors for low survival. Multiple comparisons were tested using Holm Bonferroni Sequential Correction: An EXCEL Calculator” © Justin Gaetano, 2013. *p*-values are statistically significant after this correction.

## 4. Discussion

A significant drawback of earlier research is that it either examined RNAs just in HCC associated with HBV or grouped all HCC together irrespective of the hepatitis virus implicated via tissue biopsies that are no longer an option due to their high invasiveness [19,27]. The current study evaluated the expression of circulating LINC014199, AK021443 and AF070632 in the plasma of HCV-related HCC patients aiming to examine their diagnostic and prognostic values as a noninvasive procedure. LINC014199 and AK021443 were over-expressed in the HCC patients contrasted with HCV patients and healthy controls. Our findings are in accordance with the studies that stated that HCV-HCC tissues showed different levels of both LINC014199 and AK021443 when compared to those of the normal controls [18,28]. In the current study, the expression level of mRNA AF070632 was dramatically downregulated in HCC patients than in HCV and healthy subjects. This was in line with Unfried and Fortes and, Zhang et al., who identified that AF070632 showed differential expression between early and advanced HCC suggesting that it can be deregulated not only in HCV-HCC hepatocarcinogenesis but also in the progression of the disease [18,29].

LINC01419 and AK021443 were clustered into one functional module for cell cycle regulation by the gene co-expression network analysis [18]. LINC01419 was validated to be significantly upregulated in HCV-related HCC patient samples [29] via modulating cell cycle in HCV-related HCC by helping homologous recombination (HR) repair 1 used for repairing DNA damage promoting growth, migration, invasion, and autophagy [18,30].

In addition, LINC01419 promotes HCC development and metastasis by directing EZH2-regulated RECK [30,31]. Furthermore, the downregulation of LINC01419 inhibits cell migration, invasion, and autophagy via deactivation of the PI3K/Akt1/mTOR pathway [32]. AK021443 was reported to regulate cell cycle genes and is responsible for the progression of HCC [30]. Moreover, mRNA AF070632 is mostly associated with oxidation-reduction, cofactor binding and the carboxylic acid catabolic process [18]. Interestingly, our study demonstrated that the expression levels of the circulating LINC01419 and AK021443 had higher sensitivity and specificity in distinguishing HCC patients from HCV patients and healthy controls compared to AF070632 and AFP (the most widely used diagnostic marker for HCC). Accordingly, Zhang et al. reported that LINC01419 may act as a specific biomarker for HCC as it was weakly expressed in normal liver tissue adjacent to the tumor tissues [18].

Notably, the current study revealed that LINC01419 and AK021443 were associated with advanced-stage HCC. A high plasma expression level of LINC01419 was significantly correlated with a higher Child score, while mRNA AK021443 expression level was significantly correlated with a higher viral load. On the other hand, Zhang et al., reported that LINC01419 was highly expressed in the initial stages of HCC contrasted with dysplasia [30], while Li et al. stated that mRNA AK021443 level increased in progressive phase HCC compared to healthy controls [21]. Furthermore, our study reported that LINC01419 was found to be significantly highly expressed in HCC patients with tumor size ≥ 3 cm as previously reported by Dang et al. [33] when measuring LINC01419 in HCC tissues. Additionally, LINC01419 expression levels increased in cases of portal venous invasion, which indicated more advanced HCC or the recurrence of HCC [34].

Notably, HCC Patients having high LINC01419 and AK021443 expression levels showed significantly lower overall survival as indicated by Kaplan–Meier and Log-rank survival tests. In the same context, it was reported that HCC patients having increased expression levels of AK021443 had shorter OS than those with low AK021443 expression levels, in addition, increased LINC01419 expression was significantly related to a lower OS in HCV-HCC patients [5,31].

Our study revealed that various factors involving Child Pugh classification and Portal invasion, in addition to the increased LINC01419 and AK021443 expression levels and decreased expression level of AF070632 were independent prognostic factors for OS in HCC patients. However, clinically, there are many reported limitations to the application of the Child-Pugh classification [35,36] in addition to, the subjective assessment of ascites and encephalopathy [37].

This agreed with Li et al., who reported the prognostic significance of AK02144 expression in tissues [21] and Zhang et al., whose results suggested that LINC01419 may be associated with the initiation of HCC; however, AK021443 and AF070632 may be related to the progression of HCC [18].

## 5. Conclusions

This study revealed that LINC01419 and AK021443 could be potentially used as noninvasive biomarkers for the diagnosis and prognosis of HCV-related HCC patients than mRNA AF070632 providing better insights into the molecular basis underlying hepatocarcinogenesis and new targets for limiting the progression of the disease.

## Figures and Tables

**Table 1:** Demographic and clinical data of the studied groups.

	Controls (no = 120)		Non Treated HCV (no = 194)		HCC (no = 120)		*p* Value for Test of Sig	Post Hoc Test	Effect Size
	Mean ± SD		Mean ± SD		Mean ± SD				
Age (years)	56.57 ± 6.42		56.33 ± 7.92		58.02 ± 7.32		0.126	P1 = 0.958, P2 = 0.280, P3 = 0.120	-
Sex: no, %								-	-
Male	78	65.0	136	70.1	92	76.7	0.138		
Female	42	35.0	58	29.9	28	23.3			
Smoking: no, %	24	20.0	50	25.8	40	33.3	0.062	-	-
Diabetes Mellitus: no, %	20	16.7	31	16.0	45	37.5	<0.001 *	-	-
Hypertension: no, %	17	14.2	35	18.0	48	40.0	<0.001 *	-	-
Hb (gm/dL)	13.5 ± 0.7		12.1 ± 1.8		10.6 ± 1.4		<0.001 *	P1, P2, P3 < 0.001	−0.481 [(−0.527)–(−0.431)]
TLC × 10^3^	6.8 ± 0.5		6.0 ± 1.5		5.3 ± 1.3		<0.001 *	P1, P2, P3 < 0.001	−0.359 [(−0.412)–(−0.303)]
Platelets × 10^3^	251.7 ± 28.0		164.4 ± 51.7		136.9 ± 47.2		<0.001 *	P1, P2, P3 < 0.001	−0.544 [(−0.586)–(−0.498)]
Pt	89.0 ± 9.5		82.7 ± 11.5		72.4 ± 14.7		<0.001 *	P1, P2, P3 < 0.001	−0.361 [(−0.414)–(−0.306)]
INR	1.1 ± 0.1		1.4 ± 0.3		1.4 ± 0.2		<0.001 *	P1, P2 < 0.001, P3 = 0.607	0.321 [0.264–0.376]
ALT	23 [19–25]		52.5 [38–61]		51 [44–60]		<0.001 *	P1, P2 < 0.001, P3 = 0.407	0.478 [0.428–0.524]
AST	23 [21–27]		45 [38–59]		55 [46.65]		<0.001 *	P1, P2, P3 < 0.001	0.549 [0.5047–0.591]
ALP	77 [65–91]		127 [95–187]		160 [122.7–231]		<0.001 *	P1, P2, P3 < 0.001	0.508 [0.460–0.553]
GGT	27 [25–31.7]		128 [55–155]		173 [80–217]		<0.001 *	P1, P2, P3 < 0.001	0.592 [0.550–0.631]
AFP	4.5 [3–6]		12.5 [3–31.4]		135.3 [13.5–416.3]		<0.001 *	P1, P2, P3 < 0.001	0.479 [0.430–0.526]
CEA	4.2 [2.99–5.4]		6.9 [3.9–8.9]		13.3 [6–20]		<0.001 *	P1, P2, P3 < 0.001	0.439 [0.388–0.488]
Alb	3.9 ± 0.4		4.3 ± 0.4		3.5 ± 0.6		<0.001 *	P1, P2, P3 < 0.001	−0.207 [(−0.266)–(−0.146)]
T. Bilirubin	0.6 ± 0.1		0.7 ± 0.1		1.1 ± 0.1		<0.001 *	P1, P2, P3 < 0.001	0.654 [0.617–0.688]
D. Bilirubin	0.17 ± 0.02		0.38 ± 0.12		0.50 ± 0.15		<0.001 *	P1, P2, P3 < 0.001	0.641 [0.603–0.676]
BUN	11.5 ± 1.6		11.4 ± 1.7		11.0 ± 2.9		0.059	P1 = 0.995, P2 = 0.374, P3 = 0.423	−0.071 [(−0.133)–(−0.009)]
Creatinine	0.7 ± 0.1		0.9 ± 0.2		1.1 ± 0.1		<0.001 *	P1, P2, P3 < 0.001	0.571 [0.528–0.612]
AK021443	1.01 [0.93–1.09]		2.55 [1.38–4.06]		29.34 [21.59–33.84]		<0.001 *	P1, P2, P3 < 0.001	0.696 [0.663–0.727]
LINCO01419	1.06 [1–1.12]		4.05 [1.65–30.53]		320.57 [134.4–1118.3]		<0.001 *	P1, P2, P3 < 0.001	0.688 [0.654–0.720]
AF070632	1.10 [0.98–1.3]		0.74 [0.56–0.94]		0.56 [0.46–0.65]		<0.001 *	P1, P2, P3 < 0.001	−0.553 [(−0.595)–(−0.509)]

* Significant, TLC: total leukocyte count, Pt: prothrombin time, ALP: alkaline phosphatase, T.Bil: Total. Bilirubin, D.Bil: Direct bilirubin, AFP: Alpha Fetoprotein, CEA: Carcinoembryonic antigen, Alb: albumin, BUN: Blood urea nitrogen P1: Controls vs. Non-treated HCV, P2: Controls vs. HCC, P3: Non-treated HCV vs. HCC, ANOVA and Kruskal-Wallis tests were applied. Linear trend analysis using the Jonckheere–Terpstra test was applied to detect whether there was an increasing or decreasing trend across the ordered groups. Effect size was estimated using Mann–Kendall (M–K) test to detect the presence of linear or non-linear trends (steadily increasing/decreasing or unchanging) in a series of data following Jonckheere–Terpstra (J–T) Test.

**Table 3:** Correlation between AK021443, LINC01419, AF070632 and the laboratory investigations of the studied patients’ groups.

	HCC					
	AK021443		LINC01419		AF070632	
	r_s_	*p* Value	r_s_	*p* Value	r_s_	*p* Value
Hb (gm/dL)	−0.040	0.665	−0.109	0.238	−0.033	0.717
TLC × 10^3^	**0.218**	0.017 *	0.043	0.640	−0.040	0.666
Platelets × 10^3^	**0.218**	0.017 *	−0.003	0.976	**−0.230**	0.011 *
Pt	0.004	0.964	0.044	0.633	−0.156	0.089
INR	0.073	0.430	−0.056	0.546	−0.041	0.660
ALT	**−0.291**	0.001 *	0.092	0.315	0.080	0.383
AST	**−0.224**	0.014 *	0.041	0.656	0.092	0.317
ALP	−0.057	0.533	0.034	0.712	0.099	0.281
GGT	0.065	0.484	0.084	0.359	−0.066	0.477
AFP	0.075	0.417	**0.456**	<0.001 *	**−0.228**	0.012 *
CEA	−0.019	0.840	0.101	0.271	−0.012	0.900
Alb	0.043	0.637	0.120	0.193	**−0.232**	0.011 *
T. Bilirubin	−0.028	0.765	0.089	0.333	**−0.245**	0.007 *
D. Bilirubin	**−0.224**	0.014 *	−0.110	0.233	−0.017	0.850
BUN	0.035	0.706	−0.165	0.072	0.103	0.261
Viral load	0.567	<0.001 *	**0.091**	**0.322**	**−0.121**	**0.187**
Child Pugh	0.036	0.699	**0.278**	0.002 *	**−0.184**	0.045 *
LINC01419	0.133	0.148	-	-	-	-
AF070632	−0.116	0.209	**−0.314**	<0.001 *	-	-

Hb: Hemoglobin TLC; total leukocytic count, T Bil; total bilirubin, D Bilirubin: direct bilirubin, BUN: blood urea nitrogen, AST; aspartate amino transferase, ALT; alanine aminotransferase, ALP; alkaline phosphatase, GGT: gamma glutamyl transferase, PC; prothrombin concentration, PT: prothrombin time -INR: international normalized ratios, AFP; alpha-fetoprotein_,_ CEA; carcinoembryonic antigen Alb_:_ albumin. * significant rs: Spearman correlation.

**Table 4:** Sensitivity and specificity of AK021443, LINC01419, AF070632 and AFP in diagnosis of the studied patients’ groups.

	HCC vs. Non Treated HCV			
	AK021443	LINC01419	AF070632	AFP
AUC	0.998 [0.996–1.0]	0.993 [0.987–0.999]	0.725 [0.670–0.780]	0.792 [0.735–0.849]
Cutoff point	≥12.97	≥91.84	≤0.68	>16.96
Sensitivity%	100 [96–100]	100 [96–100]	81 [72–87]	73 [64.5–81]
Specificity%	97 [91–99]	97 [91–99]	60 [51–69]	59 [52–66]
PPV%	98 [96–99]	98 [96–99]	70 [64–76]	53 [45–60]
NPV%	97 [91–99]	97 [91–99]	67 [59–74]	78 [71–85]
Accuracy	100 [96–100]	100 [96–100]	76 [66–84]	65 [59–70]

AUC: Area Under a Curve, CI: Confidence Intervals, NPV: Negative predictive value, PPV: Positive predictive value.

**Table 5:** Univariable and multivariable cox regression analysis in relation to demographic data and disease activity.

	Univariate Survival Analysis		Multivariate Analysis	
	HR [CI 95%]	*p* Value	HR [CI 95%]	*p* Value
Age (≥60)	1.71 [0.88–3.33]	0.113	-	-
Sex (male)	**3.12 [1.10–8.83]**	0.032 *	1.14 [0.35–3.70]	0.823
Co-morbidity	3.41 [0.81–14.24]	0.092	-	-
Portal invasion	**1.95 [1.0–3.80]**	0.048 *	**2.20 [1.05–4.60]**	0.037 *
CEA (High)	1.78 [0.90–3.53]	0.095	-	-
Child Pugh				
A	1.0		-	
B	4.95 [0.61–34.27]	0.137	4.24 [0.84–21.22]	0.079
C	**23.0 [3.01–175.53]**	0.002 *	**9.97 [1.96–50.58]**	0.005 *
MELD (High)	1.46 [0.75–2.83]	0.255	-	-
AFP (High)	**3.44 [1.61–7.34]**	0.001 *	**1.66 [0.67–4.10]**	**0.268**
AK021443 (High expression)	**7.42 [3.26–16.88]**	<0.001 *	**10.06 [3.36–30.07]**	<0.001 *
LINCO01419 (High expression)	**6.50 [2.52–16.75]**	<0.001 *	**4.13 [1.32–12.86]**	0.014 *
AF070632 (Low expression)	**4.11 [1.80–9.40]**	<0.001 *	**2.70 [1.07–6.81]**	0.035 *

AFP: alpha-fetoprotein, CEA; carcinoembryonic antigen, MELD; Model for End-Stage Liver Disease. * significant HR: Hazard ratio, CI: Confidence interval.

## Data Availability

The data presented in this study are available on reasonable request from the corresponding author.

## Supplementary Materials

The following supporting information can be downloaded at: https://www.mdpi.com/article/10.3390/ncrna9050062/s1, Figure S1: Co-expression genes of LINC01419 from GEPIA2 in Liver hepatocellular carcinoma; Table S1: Crosstalk relationship between them. LINC01419 and LINC01419.
