# Supplementary material for: The Potential Role of Circulating Long Miscellaneous RNAs in the Diagnosis and Prognosis of Hepatitis C Related Hepatocellular Carcinoma
Source: Noncoding RNA. 2023 Oct 11;9(5):62. doi: 10.3390/ncrna9050062 (PMC10609931; doi:10.3390/ncrna9050062)
Supplement: Supplementary file 1 [file ncrna-09-00062-s001.zip › ncrna-2605835-supplementary.pdf]

Although LINC01419 and AK021443 show the gene co-expression mode, there is no crosstalk relationship between them. The protein target of LINC01419 and the expression changes of these LINC01419-regulated targets are presented below:

Although LINC01419 and AK021443 show the gene co-expression mode (their expression increased in HCV and HCC patients in comparison to the control group), the correlation between them was found to be non-significant. Moreover, Using LncTar (http://www.cuilab.cn/lnctar) an efficient tool for predicting RNA targets of lncRNAs, LINC01419 and AK021443 are not predicted to interact with each other.

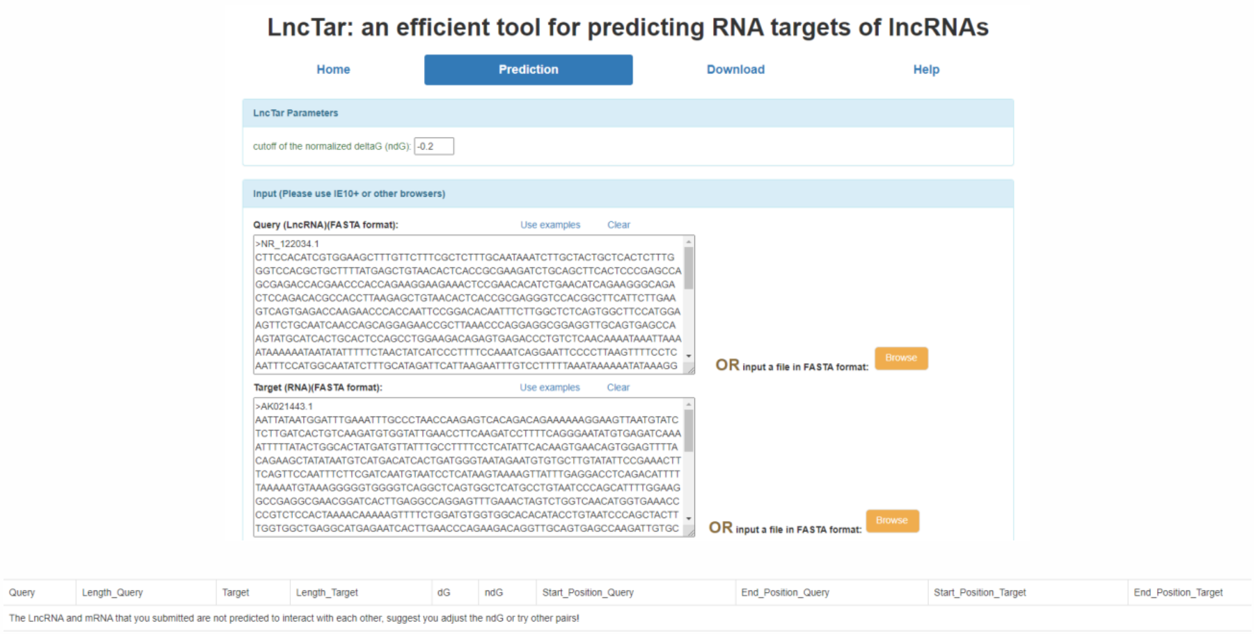

LncRNAs regulate genes expression by sponging various miRNAs. Using TarBase V.8 (https://dianalab.e-ce.uth.gr/html/diana/web/index.php?r=tarbasev8) and lncbase V 3 (https://diana.e-ce.uth.gr/lncbasev3/interactions) the target miRNAs and genes of high prediction score of LINC01419 were predicted and listed in the table below. miRpath was also used to detect the targeted pathway.

Table S1: Crosstalk relationship between LINC01419 and LINC01419

miRNA	Target genes	Pathway
hsa-let-7i-3p	STAG2, DPYSL5, COL12A1, PAQR3, HNRNPUL2, MYBPC3, USP48, ACTB, ACTB, KCNB1, KDM2A, KLHL15	cellular metabolic process, cellular protein modification process, modification of charged tRNAs
hsa-miR-106b-5p	CCND1, ZNF367, REEP3, TNKS2, ULK1, RAB22A, LDLR, G3BP1, RBBP7, TMEM127, ANKRD52, FAM117B, STYX, RUFY2, REEP5, SEMA7A, ITGB8, AHNAK, EPHA4 , LZIC	positive regulation of metabolic process, apoptotic process, protein phosphorylation
hsa-miR-130a-3p	STX6, CSF1, PSAP, PPP6R1, SMARCD2 , LDLR, ,E2F1,E2F2,E2F3,	DNA-binding transcription factor activity, RNA polymerase II-specific

hsa-miR-142-5p	AFF4, MTDH, UBE4A, EFCAB14, ANKIB1, PTPN4, RORA, DNAJB4, CHD9, GLO1	nuclear chromosome, regulation of signaling, cell development, regulation of catalytic activity
hsa-miR-148a-3p	ITGA5, CCKBR, CDKN1B, CAND1, PPP6R1, BTBD3, SBF2, DCP2 , TBL1XR1	protein metabolic process, nucleobase-containing compound metabolic process, signal transduction
hsa-miR-155-5p	ZIC3, TSHZ3, FBXO11, IL6R, DHX40, MEF2A, HBP1, IGJ, WHSC1L1	developmental process, positive regulation of cellular metabolic process, regulation of nitrogen compound metabolic process
hsa-miR-191-5p	AMMECR1L, TJP1, ZBTB34, DTD2, FUBP3, TMOD2, TLX3	developmental process, cellular nitrogen compound metabolic process, regulation of nitrogen compound metabolic process
hsa-miR-26a-5p	PDHX, CDK6, SMAD1, FGF9, PTEN, TET1, MAP3K2, HOXA9, PDCD10, GSK3B	DNA binding, gene expression, regulation of cellular component organization
hsa-miR-34a-5p	NOTCH1, MAP2K1, MET, PDGFRA, E2F3, VAMP2, MDM4, PTPN4, E2F5	system development, regulation of cellular protein metabolic process, endomembrane system
hsa-miR-454-3p	C16orf70, RNF41, ANKRD52 , PSAP, ARL6IP1, IRF1, PCNX, HPRT1, CLCN5, SMARCD2, TBL1XR1	DNA-binding transcription factor activity, metal ion binding, regulation of catalytic activity
hsa-miR-7-5p	CAND1, DDIT4, FBXO28, H2AFV, PITPNA, SETD8, ZBTB38, NUPL1	metal ion binding, cellular protein modification process, positive regulation of gene expression
hsa-miR-17-5p	STAT3 , ETV!, MMP2, TBC1D9, PKD2, SAMD12, PFN2, EIF4G2, MAP3K9	protein phosphorylation, apoptotic process, positive regulation of cellular metabolic process
hsa-miR-302c-3p	ZZNRF33, LATS2, ZBTB7A, RAB22A, MTMR4, C10orf12, KLHL15, ZNF148, ZNF800, NUFIP2	DNA-binding transcription factor activity, nuclear chromosome, nucleotide binding, establishment of localization in cell

The top 15 biological processes identified in Gene Ontology (GO) pathways revealed that mRNAs positively co-expressed with LINC01419 participated in various processes related to cancer regulation as cell proliferation, cell cycle, and migration as shown in the figure below

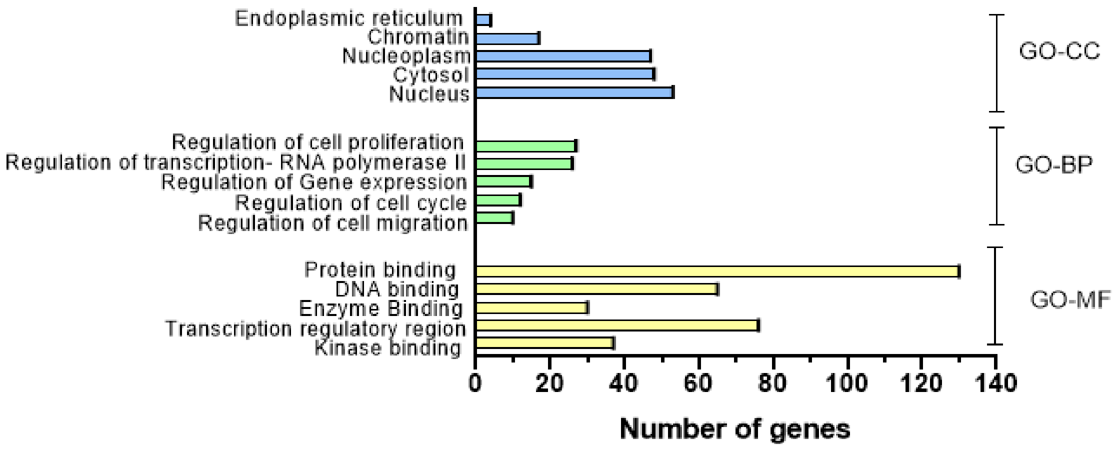

LINC01419

Moreover, LINC01419 affect the expression of various genes by other mechanisms too. To further investigate the function of LINC01419, we firstly obtained 1,000 co-expression genes of LINC01419 from GEPIA2 in LIHC. Secondly, we selected 55 mRNAs that are up or down regulated in LIHC (Liver hepatocellular carcinoma). Blue and red colors indicate the co expressed genes that are up regulated or down regulated, respectively, in LIHC as shown in the Figure below.

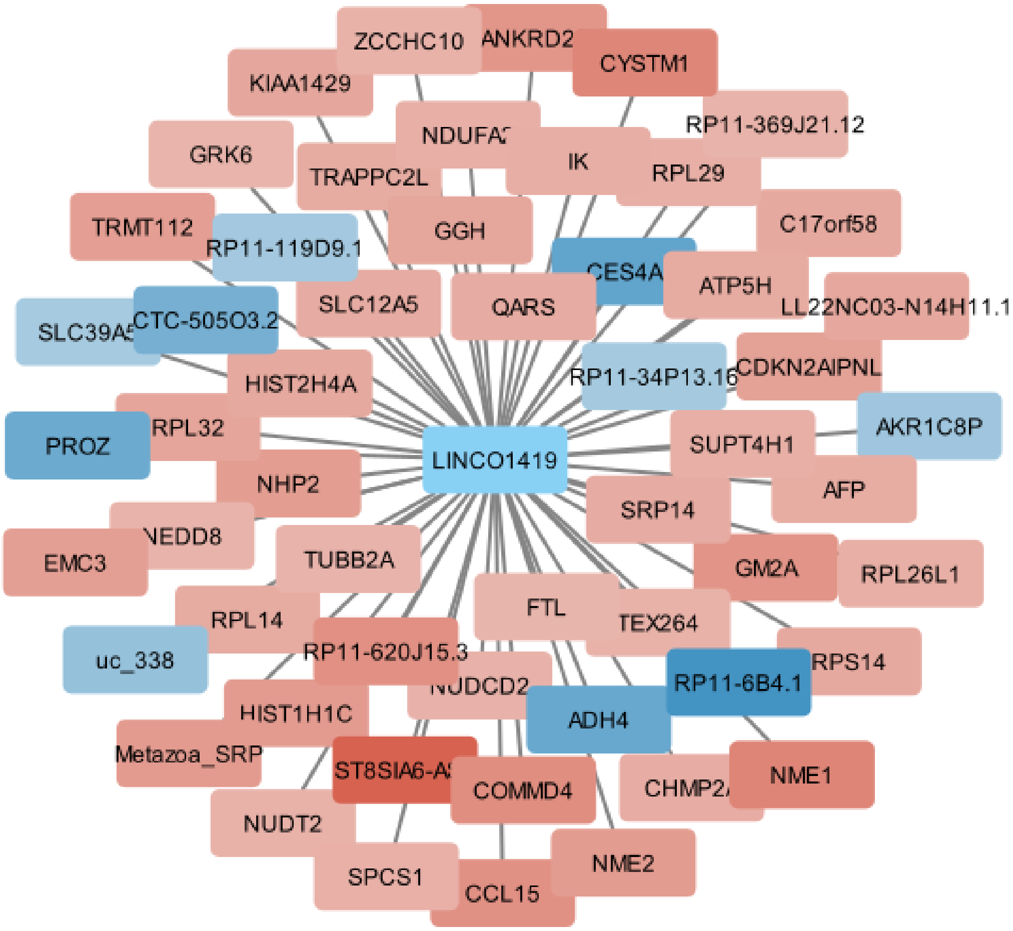

(Figure S1): Co-expression genes of LINC01419 from GEPIA2 in Liver hepatocellular carcinoma.
